# Development of an Aerosol Model of *Cryptococcus* Reveals Humidity as an Important Factor Affecting the Viability of *Cryptococcus* during Aerosolization

**DOI:** 10.1371/journal.pone.0069804

**Published:** 2013-07-23

**Authors:** Deborah J. Springer, Divey Saini, Edmond J. Byrnes, Joseph Heitman, Richard Frothingham

**Affiliations:** 1 Department of Molecular Genetics and Microbiology, Duke University Medical Center, Durham, North Carolina, United States of America; 2 Duke Human Vaccine Institute, Duke University Medical Center, Durham, North Carolina, United States of America; 3 National Institutes of Health, Office of the Director, Maryland, United States of America; University of Minnesota, United States of America

## Abstract

*Cryptococcus* is an emerging global health threat that is annually responsible for over 1,000,000 infections and one third of all AIDS patient deaths. There is an ongoing outbreak of cryptococcosis in the western United States and Canada. Cryptococcosis is a disease resulting from the inhalation of the infectious propagules from the environment. The current and most frequently used animal infection models initiate infection via liquid suspension through intranasal instillation or intravenous injection. These models do not replicate the typically dry nature of aerosol exposure and may hinder our ability to decipher the initial events that lead to clearance or the establishment of infection. We have established a standardized aerosol model of murine infection for the human fungal pathogen *Cryptococcus*. Aerosolized cells were generated utilizing a Collison nebulizer in a whole-body Madison Chamber at different humidity conditions. The aerosols inside the chamber were sampled using a BioSampler to determine viable aerosol concentration and spray factor (ratio of viable aerosol concentration to total inoculum concentration). We have effectively delivered yeast and yeast-spore mixtures to the lungs of mice and observed the establishment of disease. We observed that growth conditions prior to exposure and humidity within the Madison Chamber during exposure can alter *Cryptococcus* survival and dose retained in mice.

## Introduction

Cryptococcosis is an opportunistic fungal infection caused by *Cryptococcus* and which is a predominant cause of morbidity and mortality in immunocompromised and HIV-positive hosts [1]. *Cryptococcus* is responsible for approximately one-third of all AIDS related deaths and a more prevalent cause of HIV-related mortality in sub-Saharan Africa than tuberculosis [1]. In other developing countries infections as a result of *Cryptococcus* are second only to tuberculosis and frequently co-occur together [1]. The development of new antifungals, highly active antiviral treatment (HAART), and combination therapy regimens has increased long-term survival rates, but infections remain difficult to treat and frequently reoccur. *Cryptococcus* is now increasingly associated with a rising number of infections in seemingly healthy humans and animals worldwide [2]–[9].

Three closely related species/varieties, *Cryptococcus neoformans* var. *neoformans*, *C. neoformans* var. *grubii* and *C. gattii* are predominantly responsible for most human and animal infections. *C. neoformans* var. *grubii* is the most prevalent clinical isolate worldwide. However, *C. neoformans* var. *neoformans* is more prevalent in some regions, such as Europe. *C. gattii* is the most prevalent cause of cryptococcosis in immunocompetent hosts. However, in some regions of the world, *C. gattii* is now increasingly identified as the cause of infections in immunocompromised hosts [10]–[17]. Most cryptococcal infections result from the inhalation of infectious particles (yeast cells or spores) from the environment. Development of overt disease can be the result of acute, latent, or chronic infections. In nature *C. neoformans* var. *neoformans* is most frequently isolated from avian habitats and guano, and *C. gattii* is most frequently isolated from trees and soil. *C. neoformans* var. *grubii*, the most prevalent worldwide, has been frequently associated with avian habitats, and also trees and soil. Frequent isolation of *Cryptococcus* from plants and mating in association with plants, trees, soil, and avian guano suggest the importance of the environmental niche for both the development of infectious propagules (i.e. spores) and the maintenance of infectious reservoirs [18]–[24]. *Cryptococcus* is a prominent environmental organism, and humans are frequently exposed to its airborne infectious propagules through inhalation [25], [26]. How the environmental growth conditions and airborne infectious route influences risk and development of disease is presently unknown.

In laboratory conditions, both yeast cells and spores are effective at initiating disease but differ in size and mass [22], [23], [27]–[29]. Due to the small size of spores, it is presumed that spores are more effectively aerosolized in nature and thus more efficiently enter the bronchial tubes and alveolar spaces within the lungs [27], [29]. Additionally, spores are implicated in promoting infection because, in contrast to yeast cells, they do not require opsonization for phagocytosis by macrophages [29]. Macrophages play pivotal roles in the early host defense against *Cryptococcus* [30]. Ongoing outbreaks of *C. gattii* in the United States and Canada have renewed interest to better understand the acquisition and development of cryptococcosis.

Infections resulting from the inhalation of infectious yeast cells or spores are predominantly modeled utilizing tail-vein injection or intranasal instillation in the murine model host [31]–[33]. Although both methodologies cause morbidity and mortality, neither truly mimics the inhalation of infectious particles because these methodologies require yeast and/or spores to be administered within a suspension. The inoculation route, high dosages of cells utilized, and initial fluid solution could alter initial immune response and downstream sequela [34]–[38]. Appropriate control of cryptococcal infections depends on an intact immune system including macrophages, neutrophils, T cells, and the appropriate production and activity of cytokines [39]–[45]. Mounting evidence suggests that there are significant differences in risk to infection, development of disease, progression and host survival between the different *Cryptococcus* species [30], [37], [38], [46]–[48]. Current murine models are most frequently used but fall short of replicating the actual aerosol route of infection and may hinder our ability to decipher initial events that lead to the clearance or establishment of latent or progressive infection. Therefore these models may not accurately reflect the virulence properties of spores versus yeast, mating type **a** versus α strains, or the differences between molecular types or species [33], [49]–[53].

Aerosol infection models for *Cryptococcus* or other fungi have rarely been utilized [50], [53]–[56]. Historic applications of this approach include crude exposure from soil inoculated with *Cryptococcus,* utilization of a jet nebulizer, and infection in a Henderson Chamber [50], [54], [57]. Information extracted from the soil seeding infection was limited because infection rates and development of disease were variable, mice were exposed for multiple days, and the dosages of cells the mice were exposed to versus the dose initially retained in the lungs were not delineated. Infection initiated through the Henderson Chamber resulted in more consistent infection rates and development of disease in comparison to crude soil exposure [50]. Only 20% of mice exposed to aerosolized *Cryptococcus* basidiospores via a jet nebulizer succumbed to infection [53]. Variable infection rates and disease development were likely due to differences in the inoculum used, method of aerosolization, duration of exposure, host genotype, and strain of *Cryptococcus* utilized. These early studies were successful at initiating infection, but computer-controlled facilities were not available, *Cryptococcus* species were not yet taxonomically differentiated, and mating and the production of spores versus yeast had not been fully appreciated [50], [58].

The Madison Aerosol Chamber (College of Engineering Shop, University of Wisconsin) is now the standard apparatus for aerosol exposure of primary pathogens designated by the National Institute of Allergy and Infectious Diseases. Significant technological advancements now allow computer-controlled devices within the Madison Chamber to obtain samples pre- and post-nebulization from the exposure chamber (BioSampler), to adjust and monitor relative humidity within the chamber, and to collect pertinent data on mass and size of aerosolized cells exposed to mice [59]. These data can determine the effects of temperature, relative humidity, and the nebulization process on cells. In addition, we can calculate the dose that the mice were exposed to during a given time period, which facilitates the establishment of defined aerobiology exposure parameters for a given pathogen [59]. This study is the first contemporary application of the Madison Chamber to establish a well validated aerosol model of murine infection of *Cryptococcus*. We define the preparation of inoculum, concentration of inoculum, particle size distributions consistent with efficient aerosolization, and defined aerobiology exposure parameters for the effective and consistent delivery of aerosolized *Cryptococcus* cells to mice. A well validated inhalation model has considerable potential to facilitate novel insights into the primary exposure route of *Cryptococcus* in humans, time course of disease development, environmental conditions enhancing risk to acquisition, the role of spores and/or yeast in the infection process, and virulence of divergent mating types and genotypes which could lead to novel preventative strategies or therapeutic modalities.

## Materials and Methods

### Cryptococcus Strains, Growth Media, and Inoculum Preparation

Experiments were performed with *C. neoformans* var. *neoformans* JEC21; *C. neoformans* var. *grubii* H99α, KN99**a**, YSB119α, and KN99**a** NEO1; and *C. gattii* EJB18 (Table S1). For preliminary aerosolization tests we varied growth condition(s), exposure time, and humidity to increase the viable number of cells that mice would be exposed to within the whole-body exposure apparatus (dose presented) as determined from the viable BioSampler densities. We aimed to obtain a calculated dose presented of greater than 100 cells/mouse to maximize dose delivered and retention within the lungs. In various trials, cells were (1) grown in yeast peptone dextrose broth (YPD; 1% yeast extract, 2% peptone, 2% glucose) and prepared directly from the liquid preparation; (2) grown in YPD and subjected to natural evaporation and drying to mimic desiccation in nature, before being resuspended in water for nebulization; or (3) subcultured in YPD broth and then inoculated on agar for growth, mating, and the production of dry yeast and/or spores. Although cells were initially grown by different methods all *Cryptococcus* strains were collected and 20 mL of 1×10^8^ cell/mL suspension were used as standard inoculum density for each aerobiology exposure. To confirm the density of each cell preparation prior to nebulization (Pre-nebulization, CFU/mL) in the Madison Chamber 500 µL aliquots from every final preparation were taken and diluted on YPD agar in triplicate and colony-forming units (CFUs) were determined and compared to the expected 1×10^8^ cell/mL. In total, eleven trial experiments were performed, eight without mice, and three utilizing mice (Table S2). Broth-grown *Cryptococcus* cells were passaged twice in 250 mL flasks containing 25 mL of YPD broth with shaking at 30°C overnight (8–12 hrs). Cells were then collected by centrifugation, washed, and resuspended in 5 mL autoclaved millipore water. Cell density was determined by hemacytometer and 20 mL of a 1×10^8^ cell/mL suspension were prepared for each strain. Cells subjected to drying prior to use in the Madison Chamber were grown in identical conditions to broth grown cells, but after the initial collection by centrifugation and resuspension in 5 mL water, cells were poured into a sterile petri plate, with the lid placed slightly ajar and allowed to air dry in a BSLII hood overnight. Cells were then collected, counted with a hemacytometer, and diluted to the standard density.

In other trials Cryptococcus cells were grown on V8 agar (pH 5) or *Arabidopsis* leaf agar (20 g chopped *Arabidopsis thaliana* leaves/L, 0.1% glucose, 2% agar), which mimics plant substrates in the environment where *Cryptococcus* is known to grow and can mate. *Cryptococcus* strains were grown and subcultured twice in 10 mL YPD broth and collected by centrifugation, washed twice with 5 mL autoclaved millipore water, and resuspended in 5 mL autoclaved water. Then 100 µL of each strain was spread individually onto V8 agar or *Arabidopsis* agar plates and incubated at room temperature in the dark for two to three weeks. The colonies were scraped from the plates, washed, suspended in 5 mL water, counted with a hemacytometer, and diluted to the standard cell density.

To induce mating for the collection of yeast-spore mixtures H99α/KN99**a** or YSB119α/KN99**a** NEO1 strains were grown in combination on V8 agar (pH 5). Cells were subcultured and prepared for plating as above, but after suspension in 5 mL water, equal proportions (1.5 mL) of **a** cells were mixed with α cells and 30 to 40 individual 15 µL volumes were spotted on four to six V8 agar plates. Plates were incubated in a dark room at room temperature for three to four weeks until prolific sporulation was confirmed by light microscopy. Mating colonies (including yeast and spores) were scraped and washed with autoclaved water from each plate and resuspended in autoclaved water. The suspension was centrifuged, washed, re-suspended in 5 mL water, counted with a hemacytometer, and diluted to the standardized inoculum cell density.

### Aerosol Exposure

*Cryptococcus* aerosols were generated using a 6-jet Collison nebulizer (CN25, BGI Inc.), operated at an air-flow-rate of 13±1 lpm (19±1 PSI) and connected to a whole-body Madison Exposure Chamber (Figure S1). In trials utilizing humidified air, the nebulizer air was mixed with humidified air (37±1 lpm) as it entered the Madison Chamber. Humidified air was generated by two humidifiers connected in series (ZAB2-DT-S1-400-7, Lab Commerce Inc.). Humidity in the chamber was controlled from 30%–100% relative humidity (RH) and was measured in real-time during the aerosol exposure. Nebulized and humidified air flow rates were controlled with AeroMP (Aerosol Management Platform, Biaera Technologies, LLC) [57]. The Madison Chamber was operated under negative pressure at −13±1 inches of water column using a vacuum pump. *Cryptococcus* aerosols inside the Madison Chamber were sampled using an SKC BioSampler (SKC Inc, Model 225–9595) and aerodynamic particle sizer (APS, TSI Inc.). The start and stop operation of APS and BioSampler was controlled by AeroMP [57]. Particle size distribution by count and mass were measured by the APS at two-minute intervals for 30 seconds for the duration of the aerosol exposure. AeroMP was used to calculate the count mean aerodynamic diameter (CMAD) and mass mean aerodynamic diameter (MMAD). MMAD is used to describe a polydisperse aerosol particle size above and below which 50% of the mass of the particles is contained. The geometric standard deviation (GSD) was calculated using the particle size distributions by mass [60]. Aliquots obtained post-nebulization and from the BioSampler were dilution plated in triplicate on YPD agar, incubated at room temperature for two to four days and the average CFUs/mL was calculated. This data was utilized to determine the effect of nebulization on the viability of *Cryptococcus* cells by comparing the CFUs prior to nebulization and post-nebulization. Post-nebulization to Pre-nebulization ratio equal to or greater than one indicates 100% survival post nebulization, whereas a ratio value less than one indicates decreased viability post-nebulization. Viable aerosol density (C_a_) was used to calculate spray factor (F_s_), the ratio of viable aerosol concentration in the chamber to the starting inoculum density in the nebulizer [57], [59].

Viable aerosol concentration (C_a_) was calculated as 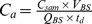 where C_sam_ is concentration in BioSampler, V_BS_ is the volume of liquid in the BioSampler after aerosol exposure, t_d_ is aerosol exposure time duration, and Q_BS_ is BioSampler air flow rate.

Spray factor was calculated as 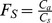 where C_a_ is viable aerosol concentration and C_S_ is nebulizer sample concentration.

The dose presented (Dp) was calculated using Guyton’s formula.


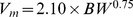
 where BW is weight of mice in grams and V_m_ is minute volume.


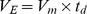
 where V_E_ is the exposure volume and t_d_ is exposure time.


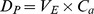
 where D_P_ is dose presented and C_a_ is the viable aerosol concentration.

A total of 15 mL sterile filtered water was used in the BioSampler to capture aerosolized samples used to determine the cell density and viability of aerosolized *Cryptococcus* inside the Madison Chamber. This density was used to calculate the dose presented (D_p_) to mice using Guyton’s formula [61]. GraphPad Prism version 6.00 for Windows (GraphPad Software, San Diego California USA) was used for data analysis to construct graphs. The P-values for linear regression analysis were calculated using the F-test. Two-way ANOVA was used to compare CFUs.

### Animals

All animal studies were conducted in the Division of Laboratory Animal Resources (DLAR) facilities at Duke University Medical Center (DUMC) and were handled according to the guidelines defined by the United States Animal Welfare Act and in full compliance with the DUMC Institutional Animal Care and Use Committee (IACUC). The aerosol infection models were reviewed and approved by the DUMC IACUC under IACUC protocol #A185-11-07. Six week old A/Jcr mice (Cat. No. 01A24, NCI-Frederick) or 10-week-old C57BL/6 mice (Cat. No. 00064, Jackson Labs) were used. Mice were acclimatized in the facility for one week prior to aerosol challenge and were housed in hermetically sealed cages at 21°C and 50% humidity with a 12 hr light/12 hr dark.

To simulate aerosol generation in nature, and with the aim of increasing the dose presented to the mice, we air dried cells grown in broth or grew cells on agar. After observing an increased dose presented, we extended the aerosol trials to include mice. For proof of principle, eight C57BL/6 mice were used to confirm that we were able to deliver and retain viable *Cryptococcus* cells within mouse lungs (dose retained). Dose retained was determined from viable CFUs from the lung homogenates. Four mice each were exposed to aerosolized *C. gattii* strain EJB18 grown in YPD broth and air-dried or cells grown on *Arabidopsis* agar. Mice were exposed to aerosolized *Cryptococcus* in the whole-body exposure chamber for 20 min. at standardized aerobiology conditions (13 lpm air-flow-rate, 19 PSI, and 70% RH). At one hour post-exposure, mice were humanely euthanized, and lung, spleen, and brain tissues were sterilely collected in a BLSII hood and homogenized in 2 mL of 1× phosphate buffered saline (PBS). Then 200 µL directly from stock and from a 1∶10 dilution was plated on YPD agar in triplicate, incubated two to four days, and CFUs were calculated to determine the doses retained in the mouse lungs. Dose retained within the lungs is a function of aerodynamic particle size, dose exposed to, and respiratory rate and volume in the animal model.

We extended our validated protocol to assess the delivery of aerosolized yeast and spores. Eight A/Jcr mice per group were exposed to aerosolized H99 (α), KN99 (**a**), or a yeast-spore mixture obtained from mated mixtures in the whole-body exposure chamber for one hour at standardized aerobiology conditions (13 lpm air flow-rate, 19 PSI, and 70% RH). Four mice per group were humanly euthanized and lung, spleen, and brain tissues were sterilely collected at one hour and three weeks post exposure and CFUs were determined as previously described.

In an additional experiment, we utilized previously constructed *C. neoformans* var. *grubii* strains YSB119 (α) and KN99**a** NEO1, which contain selectable markers for nourseothricin (NAT) or neomycin (NEO) resistance to determine if we could track the contribution of spores versus yeast throughout the aerosolization process and to the infection process (Table S2). We aimed to determine if spores survived nebulization, aerosolized, or were retained within the lungs better than yeast cells. Strains were grown individually in YPD broth under drug selection and spot plated on V8 agar (without drug addition) alone or in combination as described to induce mating. After independent culture, YSB119 and KN99**a** NEO1 cells were harvested from V8 plates and diluted to 1×10^8^ cell/mL. Then 10 mL of each cell preparation were mixed together to constitute the mixed inoculum without spores. Pre-nebulization, post-nebulization, BioSampler, and mouse tissue CFUs were determined by plating on YPD agar, YPD containing NAT (100 mg/mL), YPD containing NEO (200 mg/mL), and YPD containing both NAT and NEO. All statistical analyses were carried out using Prism (Version 5.04). Linear regression was used to assess correlations, and the Student’s T-test was used for pairwise comparisons.

## Results

### Effects of Growth Media and Humidity on Spray Factor and Viable Aerosol Concentration

**Figure 1:**
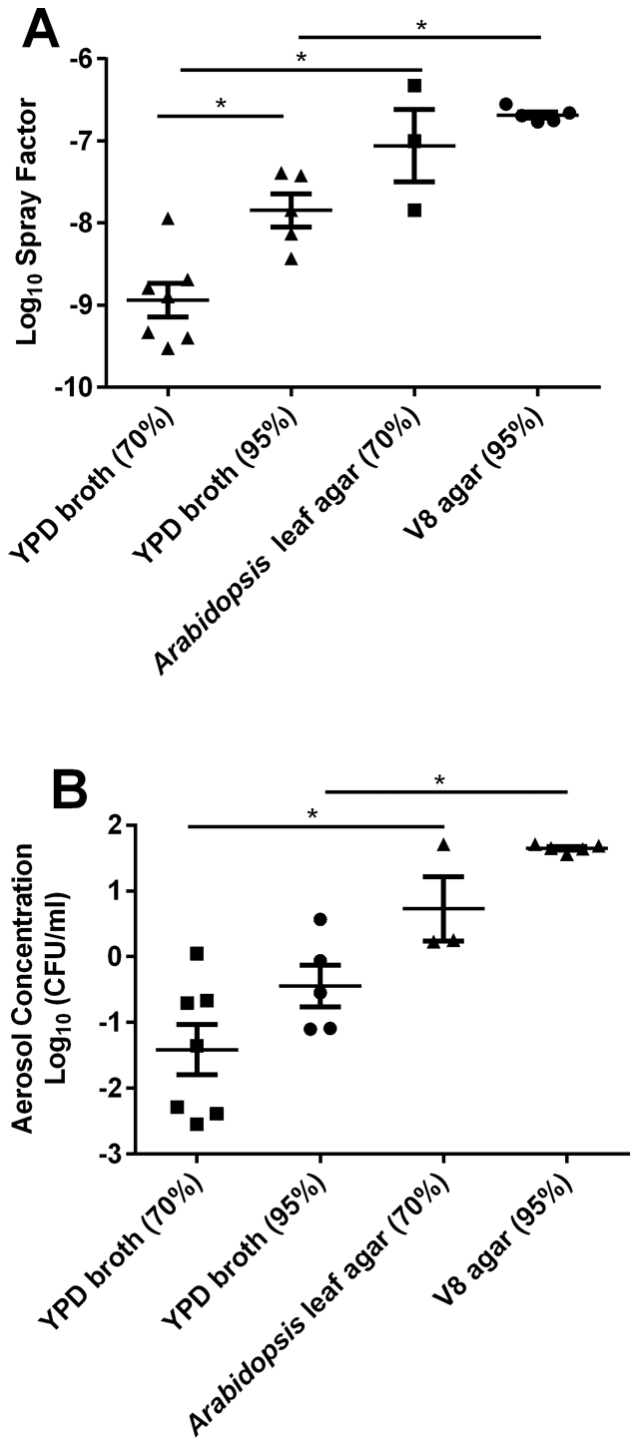
Growth on agar increases spray factor and viable aerosols for *Cryptococcus* during aerosolization in the Madison Chamber. (A) Log spray factor of *C. neoformans* var. *grubii* (H99) and *C. gattii* (EJB18) plotted in respect to growth condition and relative humidity. High humidity increases spray factor for broth-grown cells. (B) Log aerosol concentration of *C. neoformans* var. *grubii* (H99) and *C. gattii* (EJB18) plotted in respect to growth condition and relative humidity. Growth on agar increased log aerosol concentrations. Triangle, square, or circles represent growth conditions denoted in the x-axis for each individual exposure Mean values (n = 3–7, +/− SEM) are plotted. * indicates p<0.01.

**Figure 2:**
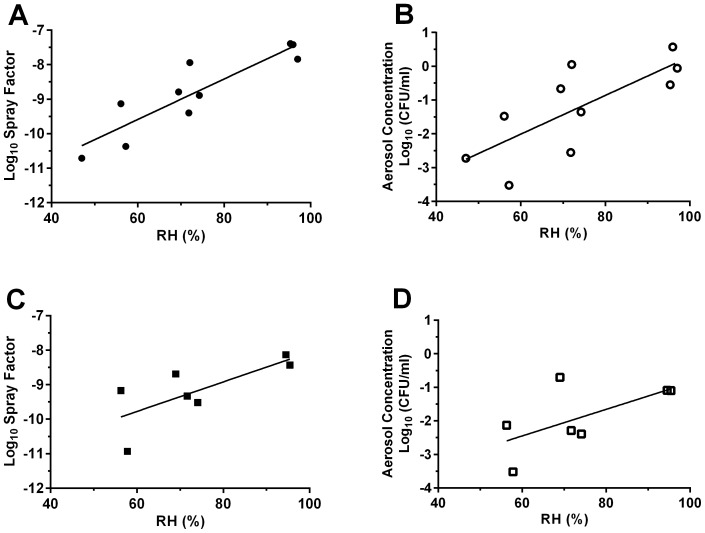
Aerosolization at high humidity increases spray factor and viable aerosols for *Cryptococcus* during aerosolization in the Madison Chamber. (A and C ) Log spray factor plotted against relative humidity for broth-grown (A) *C. neoformans* var. *grubii* H99 (p = 0.0005, R^2^ = 0.802) and (C) *C. gattii* EJB18 (p = 0.06, R^2^ = 0.534). Spray factor increases with relative humidity. (B and D) Log aerosol concentration plotted against relative humidity for broth-grown (B) *C. neoformans* var. *grubii* H99 (p = 0.011, R^2^ = 0.57) and (D) *C. gattii* EJB18 (p = 0.12, R^2^ = 0.402). Log aerosol concentration increases with relative humidity. Filled circles and squares represent *C. neoformans* var. *grubii* (H99) and open circles and squares represent *C. gattii* EJB18.

Spray factor, the ratio of viable aerosol output divided by the original suspension concentration, throughout the 11 trial experiments varied from to 4×10^−11^ to 1.4×10^−7^ (Table S2). Higher spray factor was correlated with increased dispersal and exposure to infectious particles during aerosol exposure. Increased Spray factor was observed for cells of *C. neoformans* var. *grubii* (H99) and *C. gattii* (EJB18) grown on *Arabidopsis* leaf (p = 0.003) and V8 agar (p = 0.008) in comparison to YPD broth-grown cells at 70% and 95% relative humidity (RH) (Figure 1a and Table S2). A significantly higher log spray factor for broth-grown cells was observed at 95% RH in comparison to aerosolization at 70% RH (Figure 1a, p<0.01). Growth on agar and increased humidity increased the viable aerosol concentration (BioSampler and dose presented) of *C. neoformans* var. *grubii* (H99) and *C. gattii* (EJB18) inside the Madison Chamber. At 70% (p = 0.03) and 95% RH (p = 0.005), growth on agar resulted in significantly higher values for viable aerosols than the broth-grown cells (Figure 1b). Viable BioSampler concentrations in preliminary experiments at 70% RH ranged from 3.0×10^1^ cell/mL (broth-grown) to 8.5×10^5^ cells/mL (*Arabidopsis* agar-grown). A significant positive correlation between RH and spray factor was observed for *C. neoformans* var. *grubii* (Figure 2a, p = 0.005, R^2^ = 0.802). A similar but non-significant trend was also observed in *C. gattii* (Figure 2c, p = 0.06, R^2^ = 0.532) and within a single trial with *C. neoformans* var. *neoformans* (JEC21) cells (Table S2, Trial #9 data).

Increased BioSampler concentrations were obtained with increasing humidity and increasing duration of exposure (Table S2). A direct and significant correlation between increased humidity and increased aerosol concentration was observed for H99 (Figure 2b, p = 0.011, R^2^ = 0.57). A non-statistical trend was observed for *C. gattii* EJB18 (Figure 2d, p = 0.12, R^2^ = 0.402) and *C. neoformans* var. *neoformans* (JEC21, Table S2, trial #9). Aerosol concentration of H99 and EJB18 showed greater stability at high humidity and greater variability was observed at lower humidity (Figure 2b and 2d). The highest and most consistent aerosol concentrations were obtained from growth on V8 agar media and aerosolization at 95% humidity for one hour (Figure 1 and Table S2).

### Aerosol Delivery to Mice

**Figure 3:**
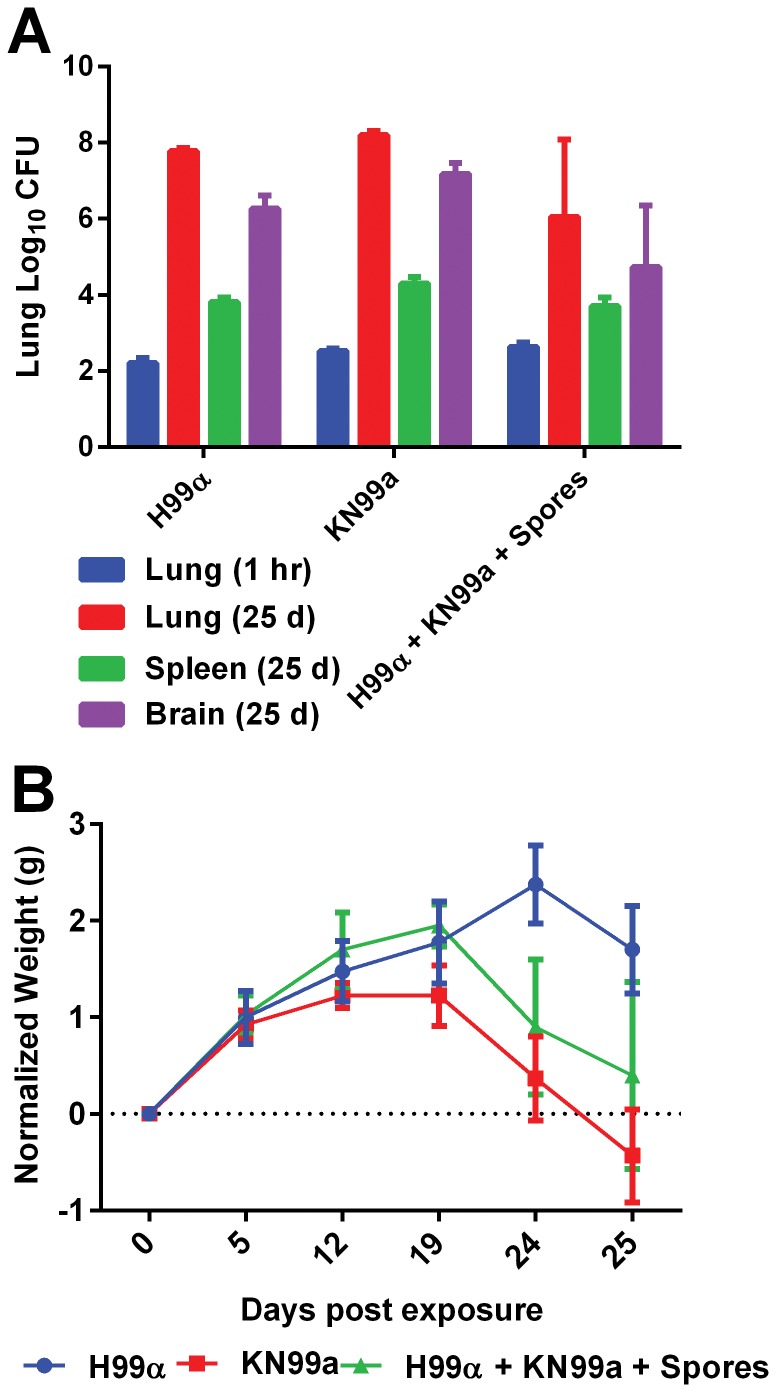
Aerosol delivery of *C. neoformans* var. *grubii* yeast and yeast-spore mixtures. (A) *C. n.*var. *grubii*, H99 (MATα), KN99**a**, and a mated mixture containing spores was effectively delivered to mice as assayed by dose retained in lungs (95% RH and 1 hour exposure). (A) Increased tissue burden, dissemination to the brain, and (B) decreased weight was observed at 24 days post-exposure and demonstrates the developmental sequelae of cryptococcosis. No *Cryptococcus* colonization was obtained from brain or spleen tissues 1 hour post exposure. No significant differences in CFUs were observed between H99 (MATα), KN99**a**, or mated mixtures (with spores) at any 1 or 25 days post exposure. Mean value (n = 3–4, +/− SEM) are plotted.

**Figure 4:**
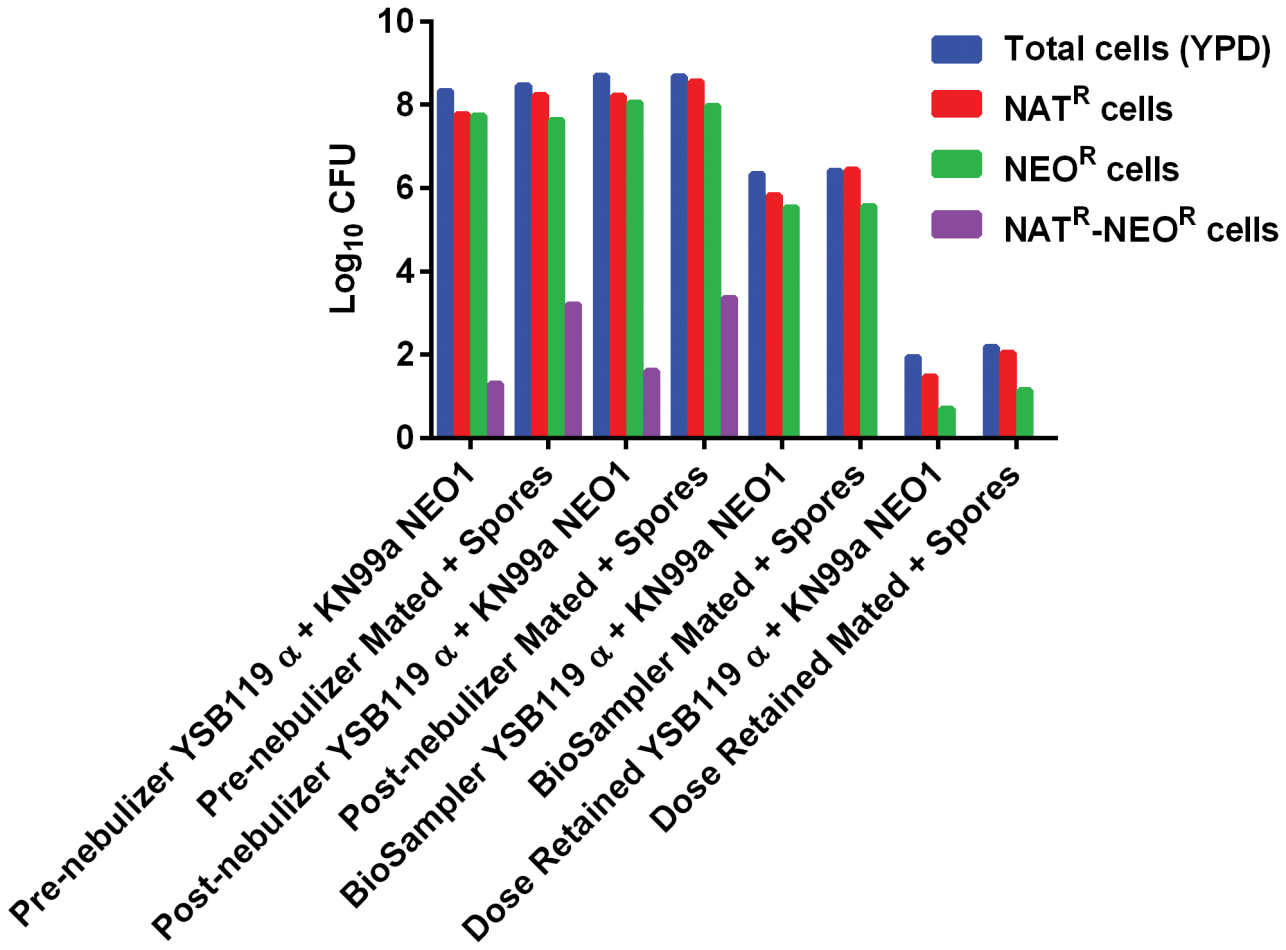
Aerosol delivery of *C. neoformans* var. *grubii* (YSB119α and KN99a NEO1) yeast and yeast-spore mixtures. Animal exposure was performed utilizing *Cryptococcus* strains containing different drug resistance markers. NAT^R^ (Red), NEO^R^ (Green), and NAT^R^+NEO^R^ (Purple) resistant colonies were obtained pre- and post- nebulization in similar proportions indicating both yeast and spores equally survive nebulization. NAT^R^+NEO^R^ resistant colonies were not observed from the BioSampler or lung tissues and we observed a net loss of total cell density (Blue) from post-nebuliztion to BioSampler, to dose presented, and to dose retained.

Mice were exposed to aerosols in three independent trial experiments (Table S2, Trial #5, #8, and #11). Delivery of aerosolized *Cryptococcus* cells to the lungs of mice was successful in all three trials. In the first preliminary mouse exposure, only 50% (EJB18, YPD-dried) to 75% (EJB18, Arabidopsis leaf agar) of mice exposed to aerosolized *C. gattii* at 70% RH for 20 minutes retained viable *C. gattii* one hour post exposure (Table S2). We observed that 100% of mice retain viable *C. neoformans* var. *grubii* cells within the lungs when mice were exposed for one hour at 95% RH. *C. neoformans* var. *grubii* H99α, KN99**a**, or the mated mixture with spores were aerosolized in individual exposures and the average doses retained were 2.07×10^2^ cells/lung, 3.52×10^2^ cells/lung, and 4.88×10^2^ cells/lung, respectively (Figure 3). Although the dose retained for the mated mixtures with spores was 1.4× (KN99**a**) and 2.4× (H99α) higher than yeast alone, this difference was not statistically significant. Spleen and brain tissues were not colonized by *Cryptococcus* one hour post-exposure (Figure 3a). Over the 24 day duration of the experimental exposure, 100% of the mice displayed patterns of weight loss and significant increase of tissue burdens (lung, brain, and spleen), and one mouse expired prior to the 24 day time-point, consistent with disease development (Figure 3b). Additionally, we used *C. neoformans* var. *grubii* strains YSB119α and KN99**a** NEO1 with drug resistance markers nourseothricin (NAT, YSB119α) or neomycin (NEO, KN99**a** NEO1) (Table S1). To consolidate exposures, we mixed YSB119α and KN99**a** NEO1 in equal portions to use as the yeast only control for the mated mixtures containing yeast and spores. Similarly, we observed a lower dosed retained in mice exposed to yeast alone (8.8×10^1^ cells/mouse) versus the yeast and spore mixtures (1.5×10^2^ cells/mouse) (Figure 4). Although dose retained for the mated mixtures with spores was 1.7× higher than yeast alone (YSB119α and KN99**a** NEO1), this difference was not statistically significant. We were able to track the presence of NAT-, NEO-, and NAT- and NEO- resistant colonies from aerosolization to post-nebulization. Doubly-drug resistant colonies represented less than 0.001% of the cells obtained pre- and post-nebulization. We also noticed a large disparity between total CFUs on YPD agar and total CFUs of NEO-resistant cells, indicating a generalized loss of the NEO resistance marker (data not shown). After 3–4 weeks incubation we observed a loss of NEO^R^ cells in the KN99**a** NEO1 strain incubated alone or in combination with YSB119α. After growth on V8 only 35% of the non-mated NEO marked parental strains retained NEO resistance. In contrast, 83% of NEO^R^ cells isolated from mating colonies retained the NEO selective marker. After observing the loss of NEO resistance in the KN99**a** NEO1 strain we confirmed that the NEO marker was episomal and not integrated by PCR, which prevented the extension of this experiment to explore the role of spores in the infection process. In summary, we obtained NAT- and NEO-resistant colonies but did not observe double-drug resistant colonies in the BioSampler or from the lung tissues of mice.

### Aerodynamic Particle Size, Concentration, and Stability during Aerosol Exposure

Aerodynamic particle characteristics mass mean aerodynamic diameter (MMAD), count mean aerodynamic diameter (CMAD), and geometric standard deviation (GSD) were determined in 8 out of the 11 independent trial exposures involving *C. neoformans* var. *neoformans, C. neoformans* var. *grubii,* and *C. gattii* (Figure S2). The MMAD distributions were similar but variable among trials and ranged from 1.0 µm–4.0 µm (Figure S2 and Table S2). However, the MMAD indicates that the particle size was consistently in the respirable range of less than 5 µm except for trial 6 in which the particle sizes ranged from 9.7 µm to 17.2 µm. The observed MMAD, CMAD, and GSD for *C. neoformans and C. gattii* were similar. MMAD of *C. gattii* ranged from 1.0 µm to 4 µm, CMAD 0.67 to 0.90, and GSD ranged from 1.2 to 2.95. The MMAD of *C. neoformans* var *grubii* ranged from 1.6 µm to 2.1 µm, CMAD 0.78 to 0.83, and GSD ranged from 1.8 to 2.74. In general, the observed MMAD was most consistent at 95% humidity. Particle size distribution by count and mass was variable and ranged up to 10 µm, but a higher concentration and mass of particles less than 5 µm were observed (Figure S3 to S6). The particle density and mass profiles for *C. gattii* (EJB18) were consistent over increased aerosolization time from 20 to 60 minutes (Figure S3). Agar grown *C. gattii* EJB18 exhibited one major particle diameter peak around 3 µm (Figure S4a, b, c) in contrast to the two peaks (∼1.5 µm and 3 µm) observed for broth grown cells (Figure S3). Agar grown *Cryptococcus* cells (Figure S4a, b, c) exhibited more consistent aerodynamic particle diameter, mass, and density profiles versus YPD-broth (Figure S4d, e) or YPD-dried cells (Figure S4f). During aerosolization of *C. neoformans* var. *grubii* (H99) particle density, mass, and size profiles remained consistent between 45% to 95% humidity (Figure S5). Broth-grown H99 cells (Figure S5) display a broad aerodynamic particle range in contrast to *C. gattii* EJB18 broth grown (Figure S3) or agar grown EJB18 cells (Figure S4). Particle mass, size, and density distributions were similar between H99 yeast cells and the mated mixture containing yeast and spores (Figure S6). The size of aerosolized *C. neoformans* var. *grubii* particles was stable during 20 minutes of exposure at 45%, 70% and 95% humidity (Figure S7a). The coefficient of variance of MMAD was 3.12% (45% RH), 3.54% (70% RH), and 3.83% (95% RH). The concentration of particles generated was more consistent at 70% and 95% RH than with 45% RH (Figure S7b). The coefficient of variance related to cell density was higher for 45% RH than 70% RH and 95% RH.

## Discussion

This is the first published report demonstrating the use of the Madison Chamber, coupled sampling port, and software for the aerosol delivery of *Cryptococcus* to mice. We demonstrated the ability to aerosolize viable *C. neoformans* and *C. gattii* cells in the respirable range and produced adequate delivery to initiate disease in the murine host. The multiplex sampling port and coupled software interface allowed measurement of viable aerosol concentration, spray factor, dose presented and particle size characteristics. These data were essential to define the aerobiology parameters for optimal and consistent delivery of *Cryptococcus* to murine lungs and to understand the characteristics of aerosolized *Cryptococcus* from generation to delivery and retention.

Preliminary trials were performed to confirm the viability of *Cryptococcus* cells post-nebulization and the quantity of viable cells in the respirable range (<10 µm) for deposition in the lungs. Initial inoculum density was maximized to 1×10^8^ cells/mL in a 20 mL volume because the viability of *Cryptococcus* post-nebulization had not been previously determined, and yeast cells were larger (up to 10 µm) than typical aerosolized pathogens [55], [59]. Our inoculum density is similar to previous reports used to initiate *C. neoformans*, *Aspergillus*, and bacterial aerosol infections, but our experiments utilized greater volume than previously reported [50], [53], [54], [56], [59]. The density of viable *Cryptococcus* yeast cells post-nebulization was equal to or greater than the inoculum density pre-nebulization. High viability post-nebulization was correlated with high spray factor. We observed a higher spray factor for *Cryptococcus* than previously reported for the primary bacterial pathogens *Yersinia pestis*, *Bacillus anthracis*, and *Mycobacterium tuberculosis* [59], [62]–[65]. *Cryptococcus* is an environmental organism that has been isolated from plants, soil, and guano. *Cryptococcus* may survive nebulization better than other organisms because in the environment, it can survive harsh conditions for long periods of time, withstanding desiccation, natural irradiation, passage through hosts, and temperature fluctuations, possibly due to its polysaccharide capsule and ability to melanize [66]. The small increases in cell density observed post-nebulization are most likely an artifact from loss of water volume during the nebulization process and not a result of increasing viability or replication. In all except one trial, MMAD was less than 5 µm and within the respirable range. In the outlier, Trial #6, MMAD ranged from 9 µm to 17 µm. We suspect that the unusual MMAD observed may be the result of inefficient desiccation of yeast cells prior to re-suspension, non-standard growth conditions, or unexplained measurement malfunction during aerosolization.

The initial two trials utilizing broth grown cells resulted in very low and inconsistent dose presented (<70 cells/mouse). Sukroongreung et al. (1989) estimated the spore load inhaled by a mouse in 15 minutes of exposure at 10^3^ to 10^6^ cells, which exceeded what we observed [53]. Effective aerosol delivery of bacteria to murine lungs has been reported with doses presented in the range of 100 to 600 cells/mouse [59]. Aerosol delivery of *Aspergillus fumigatus* conidia resulted in a dose retained of 10^3^ cells/mouse [55], [56]. We aimed to obtain a calculated dose presented greater than 100 cells/mouse to maximize dose delivered and retention within the mouse lungs because *Cryptococcus* yeast cells are larger than *Aspergillus* conidia and most bacterial cells delivered through aerosol challenge, 1 µm to 10 µm versus 0.9 µm to 2 µm, respectively [23], [67], [68]. This principle was confirmed since the observed range of MMAD, CMAD, and GSD for of aerosolized *Cryptococcus* cells was larger and more variable than previously reported for various bacteria and fungi [59], [67]–[69]. We observed a broadened aerodynamic size range for *C. gattii* comprised of two distinct peak concentrations in comparison to *C. neoformans,* which displayed a smaller size range with one single broad peak size range. These observations appear consistent with previous morphological descriptions detailing the larger and more elongated yeast and spore morphology of *C. gattii* in contrast to the more consistent and round cell morphology of *C. neoformans* [70].

In an attempt to maximize the dose presented, we grew *Cryptococcus* cells under different laboratory conditions that would more closely mimic conditions in nature, resulting in substrate-grown and/or desiccated cells. Cells were grown in YPD broth and subjected to natural desiccation overnight or grown on *Arabidopsis* agar. During the third trial, we were able to monitor relative humidity by an additional integrated package that was previously unavailable. We observed increased viable BioSampler concentrations, which resulted in a calculated dose presented that attained our predicted cut off value of >100 cells/mouse for both YPD-dried and *Arabidopsis* agar-grown cells. The increase in the calculated dose presented could be attributed to both 70% relative humidity and/or changes in the cell preparation methodology, and additional experimentation will be necessary to establish the most important factor. Both relative humidity and cell growth conditions are known to impact survival and infectivity for bacteria and viruses [71]–[78]. Additional trial exposures (without mice) were conducted to expand the aerosol model to *C. neoformans* and attempt to determine whether humidity was an important factor for a successful *Cryptococcus* aerosol model. We observed a linear increasing trend for *C. neoformans* var. *grubii* in BioSampler cell densities and dose presented that were positively correlated with increased humidity. These experiments in combination with additional experiments confirmed that increased humidity during aerosoliation increased viable aerosol and dose presented for *C. neoformans* var. *neoformans* (JEC21), *C. neoformans* var. *grubii* (H99), and *C. gattii* (EJB18). Following this finding, all aerosolization experiments were conducted at optimal humidity (RH = 95%). Humidity has been implicated to be a key factor in the viability and transmission of influenza (virus) and *Francisella tularensis* (bacteria) [77]–[79]. Low humidity is associated with increased transmission and high humidity is associated with lower transmission of influenza [77], [79]. Increased environmental isolation of *Cryptococcus gattii* has been associated with the flowering of *Eucalyptus* in Australia and during dry, hot summers in Vancouver, BC and the pacific northwestern USA [24]. The finding that humidity can influence viability and transmission of infectious propagules for fungal, viral, and bacterial pathogens suggests factors that may contribute to seasonal patterns of disease incidence.

Following the preliminary experiments, we immediately moved to conducting whole-body aerosol exposures in mice (Trial #5) upon observing a dose presented >100 cells/mouse in order to test the developmental criteria of the *Cryptococcus* aerosol exposure model. Mice were exposed to *C. gattii* cells from YPD-dried or *Arabidopsis* agar preparations. The dose presented from *Arabidopsis* agar preparations were slightly higher than those observed from the mice exposed to broth-grown and dried cells. The ability to precisely determine viable aerosols presented in the BioSampler, dose presented, and dose retained in this experiment utilizing the Madison Chamber sets these studies apart from all previously published reports on the aerosolization of *Cryptococcus*. Soil seeding and four-day exposure limited determining dose exposed and dose retained in mice that were exposed to *Cryptococcus* via soil exposure methodology. Exposure via the jet nebulizer or Henderson Chamber is much more similar to exposure via the Madison Chamber and hence is more readily comparable. Smith et al (1964) reported that mice received ∼10^4^ viable cells, but this value far exceeds CFUs obtained post mouse expiration and there is no methodology on the specifics of how this value was calculated. It is likely that what Smith et al. (1964) describes as “viable cells received” is comparable to either our BioSampler densities or viable dose presented to mice, and the former is most likely [50]. Our BioSampler densities were ∼10^3^ cells/mL to 10^5^ cells/mL which is similar to the observed “viable cells received” reported.

In our study, 50% of the mice exposed to the YPD-dried preparation had quantifiable dose retained in their lungs versus 75% of the mice exposed to the *Arabidopsis* agar preparation. These rates of initial colonization are similar to the 67% to 100% terminal infection reported by Smith et al. in 1964 and much higher than the 20% mortality rate obtained by Sukroongreung et al. (1998) [50], [53]. In contrast, aerosol delivery of *Aspergillus* to immuno-compromised mice resulted in much higher lung burden (dose retained) but resulted in only 60 to 70% mortality [55]. CFUs observed in the lungs were variable between 0–50 cells/mouse, and only a fraction of the dose presented was retained in the lungs. Some cells may be lodged in the upper respiratory system (nose, mouth, or throat), and may not immediately contribute to the dose retained. Cells deposited in the upper and lower respiratory tracts could be cleared by exhalation, killed by host defense mechanisms, or contribute to later development of infection. Actual dose presented and dose retained for each mouse was not addressed in Fu et al. (2012) but average CFUs in lung, blood, and brain tissues were determined at three and seven days post-exposure [54]. Dose retained at one-hour post-exposure in our experiments were much lower than CFUs obtained in other reports at three days, seven days, or post-expiration. In previous reports, the time-point chosen to assay dose retained varies from immediately post-exposure to 24 hrs depending on the organism. Our aim was to obtain the viable number of *Cryptococcus* cells that reached the lungs, minimizing the potential effects of killing by the host’s immunological system, replication, or dissemination of *Cryptococcus*. We defined the dose retained at the one-hour time-point because previous studies have indicated that macrophages can quickly initiate killing within two hours and significant differences in killing of *Cryptococcus* can be observed within one to four hours of *in vitro* incubation with macrophages and neutrophils [19], [40], [44]. Furthermore, previous reports have established that development of disseminated *Cryptococcus* does not often occur within hours of intranasal exposure because macrophages are implicated in the exit of *Cryptococcus* from the lungs, dissemination through the blood, passage through the blood brain barrier, and colonization of the brain [80]–[85]. We expect the initial dose retained to increase over time when *Cryptococcus* is able to survive and replicate within the host.

Additional animal exposures were performed with mated and non-mated *C. neoformans* var. *grubii* H99 (α) and KN99 (**a**) in order to determine if the low dose retained was sufficient to initiate disease development and whether the presence of spores enhanced *Cryptococcus* viability during aerosoliation, increased dose retained, or enhanced development of disease. H99α and KN99**a** yeast were aerosolized separately or from a mated mixture containing hyphal fragments, yeast, and spores. No significant differences were observed in pre-nebulization, post-nebulization, BioSampler, spray factor, or dose presented between α or **a** yeast cells alone or in combination with spores. We observed higher but non-significant dose retained in mice exposed to the mated mixtures (yeast+spores). Over time, it is possible that these small differences could be important for disease progression and the development of systemic cryptococcosis. As expected, spleen and brain tissues were not colonized by *Cryptococcus* at one hour post exposure. Over the 24-day duration of the experiment, 100% of mice displayed patterns of weight loss and significant increase of tissue burdens (lung, brain, and spleen), and one mouse expired prior to the 25-day time-point, consistent with disease development. The observed increase in CFUs, dissemination to the brain, weight loss, and death indicate progressive disease development consistent with murine infection models. We did not observe any significant differences in viable densities post-nebulization or particle size, mass, or density distribution of yeast cells or mated mixtures containing spores. Our data suggests that both yeast cells and spores are similar in their ability to survive nebulization and can share overlapping particle size, mass, and density characteristics during aerosolization. Increased relative humidity may contribute to increased viability of desiccated yeast cells post-nebulization. Our data supports the idea that in nature, both yeast cells and spore can serve as infectious particles and that risk to Cryptococcus infection may differ seasonally with varying environmental factors such as humidity. Additional research will be needed to determine if differences exist in the ability of spores versus yeast cells to traverse into the lower respiratory tract, or to enter, survive, replicate, or exit macrophages contributing to enhanced infectivity or dissemination. We demonstrated that at 95% relative humidity, we obtained 100% infection of mice, which exceeds infection efficiency reported for aerosol delivery via crude soil seeding, jet nebulizer, or Henderson apparatus [50], [53], [54].

### Conclusions

We developed a well-validated aerosol model for *Cryptococcus* utilizing the Madison Chamber and coupled instrumentation. Our data suggest a novel role of relative humidity in the viability and infectivity of *Cryptococcus.* We define inoculum growth, cell density, and aerobiology delivery parameters for consistent delivery of low numbers of *Cryptococcosis* to murine lungs.

## Supporting Information

Figure S1 **Madison Chamber and whole body exposure system.** (A and C) Overview of the Aerosol exposure system contained within the Class III BioSafety Cabinet (2). (B) The Madison Chamber (1) is attached to a Class III BioSafety Cabinet (2). AeroMP (3) collects data from the chamber pressure sensor (3), Relative humidity and temperature probe (4), and (5) Aerodynamic particle sizer (APS). AeroMP (3) also controls the nebulizer flow, dilution airflow (6), humidifier, BioSampler, and APS. Mice were exposed in a whole body exposure apparatus (7) housed within the Madison Chamber. Directional flow is delineated with red arrows, Collison nebulizer (8), and Impinger (9).
(PNG)

Figure S2 **Mass Median Aerodynamic Diameter (MMAD) of** ***Cryptococcus*** **during aerosolization in the Madison Chamber.** MMAD of all trials appeared relatively consistent between 1.5 µm and 4.0 µm except for trial #6. MMAD for trial #11 was not plotted because APS was not functional. No significant differences were observed in the MMAD of *C. neoformans* var. *grubii* (H99) and *C. gattii* (EJB18).
(TIF)

Figure S3 **Aerodynamic particle characteristics of** ***C. gattii*** **remain consistent during aerosolization over different lengths of time.** Trial #2 (A) EJB18 30 min.; (B) EJB18 45 min.; (C) EJB18 60 min. YPD-broth grown and RH undetermined. Particle mass (open blue triangle) and particle density (open red circle) plotted respective to particle diameter.
(TIF)

Figure S4 **Aerodynamic particle characteristics of** ***C. gattii*** **during aerosolization.** Agar grown cells display more consistent particle size, mass, and density in comparison to cells grown in YPD-broth. The aerodynamic properties of cells grown in broth with drying are more consistent then cells prepared for aerosolization without drying. (A) EJB18 *Arabidopsis* agar, Trial #3; (B) EJB18 *Arabidopsis* agar, Trial #3; (C) EJB18 *Arabidopsis* agar, Trial #5; (D) EJB18 YPD-dried; Trial # 5 (E) EJB18 YPD-dried; Trial #10 (F) EJB18 YPD-broth 45% RH. Particle mass (open blue triangle) and particle density (open red circle) plotted respective to particle diameter.
(TIF)

Figure S5 **Aerodynamic particle characteristics of** ***C. neoformans*** **var.** ***grubii*** **remain stable during aerosolization at different relative humidity.** Trial #7 YPD-broth grown (A) H99α 45% RH; (B) H99α 70% RH; (C) H99α 95% RH. Particle mass (open blue triangle) and particle density (open red circle) plotted respective to particle diameter.
(TIF)

Figure S6 **Aerodynamic particle characteristics of** ***C. neoformans*** **var.** ***grubii*** **yeast cells and mated mixtures containing spores are similar.** (E) H99α, Trial #8; (F) Mated mixture H99α+KN99**a**+Spores, Trial #8. Particle mass (open blue triangle) and particle density (open red circle) plotted respective to particle diameter. Cells were V8 agar grown and aerosolized at 95% relative humidity.
(TIF)

Figure S7 ***Cryptococcus*** **particle size stability and density during aerosol exposure.** Aerodynamic mass distribution was measured using the Aerodynamic Particle Sizer (APS) connected to the Madison Chamber. Sampling was done for 30 seconds, every 2 minutes for 20 minutes during Trial #10a. During aerosolization MMAD (A) and aerosol concentration (B) of *C. neoformans* var. *grubii* (H99) were measured. MMAD remains consistent when aerosolized at different relative humidity. Particle density changes over the 20 min exposure when aerosolized at 45% humidity but remains constant when aerosolized at 70% and 95% relative humidity. Circle 45% RH, square 70% RH, triangle 95% RH.
(TIF)

Table S1 **Strains utilized in this study.**
(DOC)

Table S2 **Aerosol experiments.** Eleven independent aerosol trial experiments were performed to optimize viable aerosol, dose presented and dose retained in the mouse model host. Three independent trials included mice (Trials #5, #8, and #11).
(DOC)
