# Supplementary figures and images for: Development of an Aerosol Model of Cryptococcus Reveals Humidity as an Important Factor Affecting the Viability of Cryptococcus during Aerosolization
Source: PLoS One. 2013 Jul 23;8(7):e69804. doi: 10.1371/journal.pone.0069804 (PMC3720958; doi:10.1371/journal.pone.0069804)

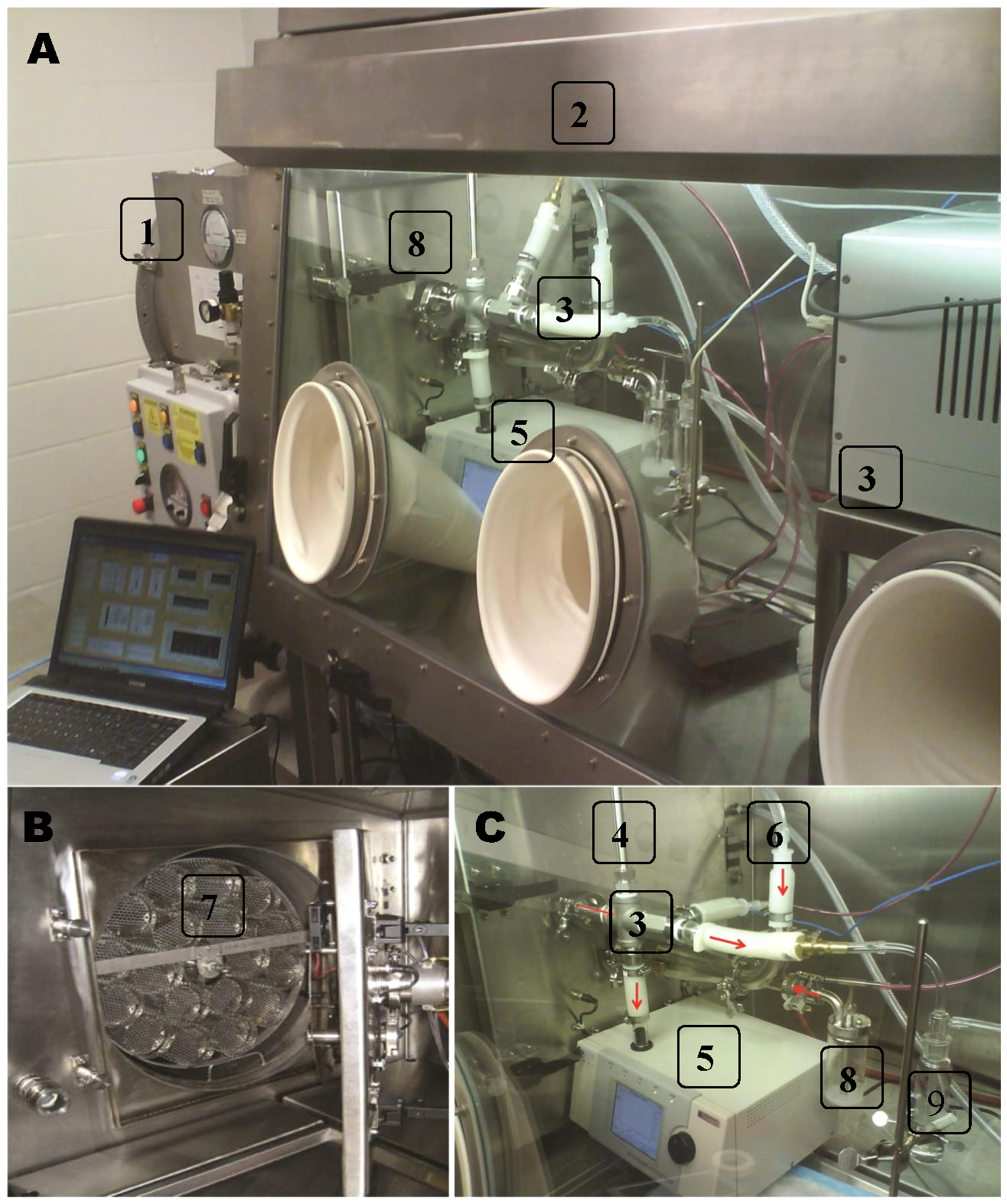

Supplement: Figure S1 — Madison Chamber and whole body exposure system. (A and C) Overview of the Aerosol exposure system contained within the Class III BioSafety Cabinet (2). (B) The Madison Chamber (1) is attached to a Class III BioSafety Cabinet (2). AeroMP (3) collects data from the chamber pressure sensor (3), Relative humidity and temperature probe (4), and (5) Aerodynamic particle sizer (APS). AeroMP (3) also controls the nebulizer flow, dilution airflow (6), humidifier, BioSampler, and APS. Mice were exposed in a whole body exposure apparatus (7) housed within the Madison Chamber. Directional flow is delineated with red arrows, Collison nebulizer (8), and Impinger (9). (PNG) [file pone.0069804.s001.png]
