# Supplementary material for: Development of an Aerosol Model of Cryptococcus Reveals Humidity as an Important Factor Affecting the Viability of Cryptococcus during Aerosolization
Source: PLoS One. 2013 Jul 23;8(7):e69804. doi: 10.1371/journal.pone.0069804 (PMC3720958; doi:10.1371/journal.pone.0069804)
Supplement: Table S1 — Strains utilized in this study. (DOC) [file pone.0069804.s008.doc]

| **Supplemental Table 1. Strains used in this study.** | | | |
| --- | --- | --- | --- |
| **Strain** | **Species** | **Genotype** | **Reference** |
| EJB18 | *C. gattii* | *MAT*α | Byrnes et al. 2009. Infectious Dis. 199:1081-1086. |
| H99 | *C. neoformans* var. *grubii* | *MAT*α | Nielsen et al. 2005. Infect Immun. 73:4922-4933. |
| KN99**a** | *C. neoformans* var. *grubii* | *MAT***a** congenic with H99 | Nielsen et al. 2005. Infect Immun. 73:4922-4933 |
| YSB119 | *C. neoformans* var. *grubii* | *MAT*α *aca1*Δ::*NAT-STM#43 ura5 ACA1-URA5* | Bahn et al. 2004. Eukaryotic Cell. 3:1476-1491 |
| KN99**a** NEO1 | *C. neoformans* var. *grubii* | *KN99***a***: Episomal NEO; MAT***a** | Lab Stock #6751 |
| JEC21 | *C. neoformans* var. *neoformans* | *MAT*α | (Sanfelice) Vuillemin, anamorph (ATCC MYA­565D­5) |
