# Supplementary material for: Development of an Aerosol Model of Cryptococcus Reveals Humidity as an Important Factor Affecting the Viability of Cryptococcus during Aerosolization
Source: PLoS One. 2013 Jul 23;8(7):e69804. doi: 10.1371/journal.pone.0069804 (PMC3720958; doi:10.1371/journal.pone.0069804)
Supplement: Table S2 — Aerosol experiments. Eleven independent aerosol trial experiments were performed to optimize viable aerosol, dose presented and dose retained in the mouse model host. Three independent trials included mice (Trials #5, #8, and #11). (DOC) [file pone.0069804.s009.doc]

| **Supplemental Table 2. Experimental conditions and results for each trial.*** | | | | | | | | | | | | | | |
| --- | --- | --- | --- | --- | --- | --- | --- | --- | --- | --- | --- | --- | --- | --- |
| **Trial** | **Cryptococcal strain** | **Growth Condition** | **Pre-Nebuliz-ation**  **(CFU/mL)** | **Neb-uliz-ation Time (min)** | **Post-Nebuliz-ation (CFU/mL)** | **Post/Pre Ratio** | **Bio-Sampler (CFU/mL)** | **Aerosol (CFU/ml)** | **Spray Factor** | **Mass Median Aero-dynamic Dia-meter (µm)** | **Geo-metric Standard Deviation** | **Dose Presented (CFU/ mouse)** | **Dose Retained (CFU/ mouse)** | **Humi-dity (RH)** |
| 1 | EJB18 | YPD-broth | 1.00E+06 | 20 | 1.70E+06 | 1.70 | 7.00E+02 | 0.04 | 4.20E-08 | 1.60 | 1.92 | 10.0 | ND | NA |
| 1 | EJB18 | YPD-broth | 1.00E+07 | 20 | 8.00E+06 | 0.80 | 6.50E+02 | 0.04 | 3.90E-09 | 2.46 | 2.74 | 6.8 | ND | NA |
| 1 | EJB18 | YPD-broth | 1.00E+08 | 20 | 1.60E+08 | 1.60 | 3.00E+01 | 0.002 | 1.80E-11 | 4.07 | 2.95 | 0.2 | ND | NA |
| 2 | EJB18 | YPD-broth | 1.00E+08 | 30 | 9.50E+07 | 0.95 | 1.00E+02 | 0.004 | 4.00E-11 | 3.79 | 3.93 | 1.43 | ND | NA |
| 2 | EJB18 | YPD-broth | 1.00E+08 | 45 | 1.21E+08 | 1.21 | 5.00E+01 | 0.001 | 1.33E-11 | 2.29 | 2.55 | 0.52 | ND | NA |
| 2 | EJB18 | YPD-broth | 1.00E+08 | 60 | 2.17E+08 | 2.17 | 1.04E+04 | 0.21 | 2.08E-09 | 2.64 | 2.74 | 68 | ND | NA |
| 3 | EJB18 | YPD-dried | 1.60E+07 | 20 | 2.30E+07 | 1.44 | 3.12E+03 | 0.19 | 1.17E-08 | 2.12 | 2.21 | 66 | ND | 70.29 |
| 3 | EJB18 | Arabidopsis agar | 1.80E+07 | 20 | 3.16E+07 | 1.76 | 2.98E+04 | 1.79 | 9.93E-08 | 3.05 | 4.22 | 355 | ND | 68.78 |
| 3 | EJB18 | Arabidopsis agar | 3.67E+07 | 20 | 1.16E+08 | 3.16 | 2.81E+04 | 1.69 | 4.59E-08 | 2.64 | 3.64 | 336 | ND | 69.25 |
| 4 | EJB18 | Arabidopsis agar | 7.26E+08 | 20 | 4.96E+07 | 0.07 | 8.01E+03 | 0.48 | 6.62E-10 | 3.28 | 1.24 | 153 | ND | 69.82 |
| 4 | EJB18 | Arabidopsis agar | 7.26E+08 | 20 | 5.49E+07 | 0.08 | 6.93E+03 | 0.42 | 5.73E-10 | 3.28 | 1.33 | 132 | ND | 68.82 |
| 4 | EJB18 | YPD-dried A | 1.52E+08 | 20 | 1.92E+08 | 1.26 | 3.75E+05 | 22.50 | 1.48E-07 | 3.52 | 4.22 | 7,170 | ND | 69.5 |
| 4 | EJB18 | YPD-dried B | 1.06E+08 | 20 | 2.15E+08 | 2.03 | 9.00E+03 | 0.54 | 5.09E-09 | 3.79 | 4.22 | 267 | ND | 68.84 |
| 5 | EJB18 | YPD-dried | 7.30E+07 | 20 | 8.20E+07 | 1.12 | 7.70E+03 | 0.46 | 6.33E-09 | 1.04 | 1.55 | 138 | 0 to 53.3 | 71.83 |
| 5 | EJB18 | Arabidopsis agar | 9.50E+07 | 20 | 1.09E+08 | 1.15 | 8.50E+05 | 51.00 | 5.37E-07 | 2.46 | 1.24 | 16,500 | 0 to 26.3 | 71.73 |
| 6 | EJB18 | YPD-dried | 7.26E+08 | 20 | 8.01E+08 | 1.10 | 2.31E+03 | 0.14 | 1.91E-10 | 17.20 | 2.56 | 39 | ND | 69.25 |
| 6 | EJB18 | YPD-broth | 9.70E+07 | 20 | 1.48E+08 | 1.53 | 3.30E+03 | 0.20 | 2.04E-09 | 12.90 | 2.06 | 56 | ND | 68.99 |
| 6 | H99 | YPD-dried | 9.70E+08 | 20 | 3.51E+09 | 3.62 | 3.01E+03 | 0.18 | 1.86E-10 | 12.00 | 1.78 | 51 | ND | 69.49 |
| 6 | H99 | YPD-broth | 1.32E+08 | 20 | 1.77E+08 | 1.34 | 3.60E+03 | 0.22 | 1.64E-09 | 9.65 | 2.74 | 61 | ND | 69.44 |
| 7 | H99 | YPD-broth | 9.86E+07 | 20 | 2.41E+08 | 2.44 | 3.17E+01 | 0.002 | 1.93E-11 | 1.60 | 1.92 | 0.5 | ND | 47.02 |
| 7 | H99 | YPD-broth | 9.86E+07 | 20 | 1.89E+08 | 1.92 | 1.87E+04 | 1.12 | 1.14E-08 | 1.72 | 1.92 | 329 | ND | 72.05 |
| 7 | H99 | YPD-broth | 9.86E+07 | 20 | 1.58E+08 | 1.60 | 6.24E+04 | 3.74 | 3.80E-08 | 1.60 | 1.78 | 1,130 | ND | 95.94 |
| 8 | H99 | V8 agar (pH5) | 2.12E+08 | 60 | 1.06E+09 | 5.00 | 1.80E+06 | 36.00 | 1.70E-07 | 1.98 | 2.05 | 39,600 | 207 ± 73 | 96.96 |
| 8 | KN99**a** | V8 agar (pH5) | 2.07E+08 | 60 | 6.00E+08 | 2.90 | 2.24E+06 | 44.80 | 2.16E-07 | ND | ND | 41,000 | 352 ± 110 | 95.17 |
| 8 | H99 x KN99**a** +Spores | V8 agar (pH5) | 1.77E+08 | 60 | 1.21E+09 | 6.84 | 2.47E+06 | 49.40 | 2.79E-07 | 2.13 | 2.37 | 45,200 | 488 ± 278 | 95.49 |
| 9 | EJB18 | YPD-broth | 1.02E+08 | 20 | 1.16E+07 | 0.11 | 1.20E+03 | 0.07 | 6.49E-10 | NA | NA | 25 | ND | 49.43 |
| 9 | EJB18 | YPD-broth | 8.05E+07 | 20 | 1.07E+07 | 0.13 | 2.09E+03 | 0.10 | 1.25E-09 |  |  | 38 | ND | 67.1 |
| 9 | EJB18 | YPD-broth | 9.10E+07 | 20 | 7.67E+06 | 0.08 | 3.69E+03 | 0.19 | 2.11E-09 |  |  | 73 | ND | 74.55 |
| 9 | JEC21 | YPD-broth | 4.50E+08 | 20 | 6.80E+06 | 0.02 | 2.83E+01 | 0.001 | 2.90E-12 |  |  | 0.5 | ND | 47.07 |
| 9 | JEC21 | YPD-broth | 3.60E+08 | 20 | 6.57E+06 | 0.02 | 7.89E+02 | 0.04 | 1.01E-10 |  |  | 14 | ND | 68.08 |
| 9 | JEC21 | YPD-broth | 5.90E+08 | 20 | 6.63E+06 | 0.01 | 1.18E+03 | 0.05 | 9.17E-11 |  |  | 21 | ND | 74.22 |
| 10A | H99 | YPD-broth | 7.57E+06 | 20 | 4.70E+06 | 0.67 | 5.00E+00 | 0.0003 | 4.29E-11 | 1.56 | 1.83 | 0.11 | ND | 57.23 |
| 10A | H99 | YPD-broth | 7.57E+06 | 20 | 6.00E+06 | 0.86 | 4.67E+01 | 0.0028 | 4.00E-10 |  |  | 1.07 | ND | 71.82 |
| 10A | H99 | YPD-broth | 7.57E+06 | 20 | 8.60E+06 | 1.23 | 4.72E+03 | 0.28 | 4.04E-08 |  |  | 108.17 | ND | 95.36 |
| 10A | EJB18 | YPD-broth | 9.05E+06 | 20 | 1.12E+07 | 1.02 | 1.23E+02 | 0.01 | 6.73E-10 | 1.49 | 1.79 | 2.83 | ND | 56.29 |
| 10A | EJB18 | YPD-broth | 9.05E+06 | 20 | 1.18E+07 | 1.07 | 8.50E+01 | 0.01 | 4.64E-10 |  |  | 1.95 | ND | 71.66 |
| 10A | EJB18 | YPD-broth | 9.05E+06 | 20 | 3.96E+07 | 3.60 | 1.35E+03 | 0.08 | 7.34E-09 |  |  | 30.85 | ND | 94.53 |
| 10B | H99 | YPD-broth | 5.86E+07 | 20 | 4.55E+07 | 0.89 | 5.57E+02 | 0.03 | 7.34E-10 | NA | NA | 12.77 | ND | 56.07 |
| 10B | H99 | YPD-broth | 5.86E+07 | 20 | 3.46E+07 | 0.67 | 7.38E+02 | 0.04 | 1.28E-09 |  |  | 16.93 | ND | 74.26 |
| 10B | H99 | YPD-broth | 5.86E+07 | 20 | 6.15E+07 | 1.20 | 1.47E+04 | 0.88 | 1.43E-08 |  |  | 336.66 | ND | 97.00 |
| 10B | EJB18 | YPD-broth | 7.25E+06 | 20 | 2.56E+07 | 2.41 | 5.00E+00 | 0.00 | 1.17E-11 |  |  | 0.11 | ND | 57.80 |
| 10B | EJB18 | YPD-broth | 7.25E+06 | 20 | 1.36E+07 | 1.28 | 6.83E+01 | 0.00 | 3.03E-10 |  |  | 1.57 | ND | 74.09 |
| 10B | EJB18 | YPD-broth | 7.25E+06 | 20 | 2.13E+07 | 2.01 | 1.31E+03 | 0.08 | 3.69E-09 |  |  | 30.08 | ND | 95.42 |
| 11 | YSB119+KN99**a** NEO1 | V8 agar (pH5) | 2.13E+08 | 60 | 4.97E+08 | 2.33 | 2.17E+06 | 36 | 1.70E-07 | NA | NA | 1.66E+04 | 88 ± 63 | 95.25 |
| 11 | YSB119-KN99**a** NEO1+Spores | V8 agar (pH5) | 2.89E+08 | 60 | 4.83E+08 | 1.67 | 2.40E+06 | 40 | 1.38E-07 | NA | NA | 1.98E+04 | 153 ± 78 | 93.25 |

*The table summarizes the experimental conditions for each trial. Each trial consisted of several independent aerosolizations of a liquid cryptococcal inoculum. The pre-nebulization and post-nebulization concentrations represent the viable fungal colony-forming units in the liquid inoculum preparation before and after the nebulization process. A Biosampler was used to capture particles from the aerosol chamber during nebulization. The viable aerosol concentration was calculated from the Biosampler concentration. The spray factor is the ratio between the aerosol concentration and the pre-nebulization liquid inoculum preparation. The mass mean aerodynamic diameter (MMAD) was determined by sampling the aerosol in the chamber with an aerodynamic particle sizer. The geometric standard deviation (GSD) is based on the distribution of aerodynamic sizes. The dose presented represents an estimate of the viable fungi inhaled by an exposed mouse. It is calculated on the basis of the aerosol concentration, duration of aerosol exposure, and minute ventilatory volume. The minute ventilatory volume was estimated based on the average weight of the exposed mice with a weight of 19 grams used when no mice were exposed. The dose retained was determined by cultures from lung homogenates obtained 1 hour after the aerosol exposure in groups of 4 to 7 mice. The dose retained is shown as mean ± SD, except in experiment 5 where the range is shown since the data were not normally distributed.
